# Interplay of Matrix Stiffness and c-SRC in Hepatic Fibrosis

**DOI:** 10.3389/fphys.2015.00359

**Published:** 2015-12-02

**Authors:** Jan Görtzen, Robert Schierwagen, Jeanette Bierwolf, Sabine Klein, Frank E. Uschner, Peter F. van der Ven, Dieter O. Fürst, Christian P. Strassburg, Wim Laleman, Jörg-Matthias Pollok, Jonel Trebicka

**Affiliations:** ^1^Department of Internal Medicine I, University of BonnBonn, Germany; ^2^Department of General, Visceral, Thoracic, and Vascular Surgery, University of BonnBonn, Germany; ^3^Department of Molecular Cell Biology, Institute for Cell Biology, University of BonnBonn, Germany; ^4^Department of Internal Medicine, University Hospital GasthuisbergLeuven, Belgium; ^5^Faculty of Health Sciences, University of Southern DenmarkOdense, Denmark

**Keywords:** RhoA, SRC, liver fibrosis, matrix stiffness, hepatic stellate cells, PP2

## Abstract

**Introduction:** In liver fibrosis activation of hepatic stellate cells (HSC) comprises phenotypical change into profibrotic and myofibroplastic cells with increased contraction and secretion of extracellular matrix (ECM) proteins. The small GTPase RhoA orchestrates cytoskeleton formation, migration, and mobility via non-receptor tyrosine-protein kinase c-SRC (cellular sarcoma) in different cells. Furthermore, RhoA and its downstream effector Rho-kinase also play a crucial role in hepatic stellate cells and hepatic fibrogenesis. Matrix stiffness promotes HSC activation via cytoskeleton modulation. This study investigated the interaction of c-SRC and RhoA under different matrix stiffness conditions.

**Methods:** Liver fibrosis was induced in rats using bile duct ligation (BDL), thioacetamide (TAA) or carbon tetrachloride (CCl_4_) models. mRNA levels of *albumin, PDGF-R, RHOA, COL1A1*, and α*SMA* were analyzed via qRT-PCR. Western Blots using phospho-specific antibodies against p-c-SRC418 and p-c-SRC530 analyzed the levels of activating and inactivating c-SRC, respectively. LX2 cells and hepatocytes were cultured on acrylamide gels of 1 and 12 kPa or on plastic to mimic non-fibrotic, fibrotic, or cirrhotic environments then exposed to SRC-inhibitor PP2. Overexpression of RhoA was performed by transfection using RhoA-plasmids. Additionally, samples from cirrhotic patients and controls were collected at liver transplantations and tumor resections were analyzed for RhoA and c-SRC protein expression by Western Blot.

**Results:** Transcription of albumin and RhoA was decreased, whereas transcription and activation of c-SRC was increased in hepatocytes cultured on 12 kPa compared to 1 kPa gels. LX2 cells cultured on 12 kPa gels showed upregulation of *RHOA, COL1A1*, and α*SMA* mRNA levels. Inhibition of c-SRC by PP2 in LX2 cells led to an increase in *COL1A1* and α*SMA* most prominently in 12 kPa gels. In LX2 cells with RhoA overexpression, c-SRC inhibition by PP2 failed to improve fibrosis. RhoA expression was significantly elevated in human and experimental liver fibrosis, while c-SRC was inactivated.

**Conclusions:** This study shows that c-SRC is inactive in activated myofibroblast-like HSC in liver cirrhosis. Inactivation of c-SRC is mediated by a crosstalk with RhoA upon hepatic stellate cell activation and fibrosis progression.

## Introduction

End-stage liver disease is characterized by fibrosis and loss of organ function, and is one of the leading causes of death worldwide (World Health Organisation, 2002; National Institute on Alcohol Abuse and Alcoholism, 2014). In chronic liver injury, hepatic stellate cells (HSC) get activated, proliferate, and migrate within liver tissue (Rockey, 1997). Moreover, activated HSCs are the major contributors to liver fibrogenesis by extracellular matrix (ECM) synthesis (Mederacke et al., 2013). The small GTPase RhoA is a master regulator protein and mediates HSC activity and motility by signaling downstream to effectors Rho-kinase (ROCK) or mDia1 (Thumkeo et al., 2013). In liver fibrosis, upregulation of the RhoA/ROCK axis leads to increased vascular contractility and portal pressure (Hennenberg et al., 2006; Trebicka et al., 2007). The RhoA/ROCK signaling exerts these effects via activated HSCs (Trebicka et al., 2010). Inhibition of RhoA/ROCK activity in liver fibrosis reduces portal pressure and attenuates hepatic fibrosis by induction of HSC senescence (Trebicka et al., 2010; Klein et al., 2012a,b). Besides the RhoA/ROCK axis, RhoA also interacts with the cytosolic tyrosine kinase c-SRC via mDia1 (Yamana et al., 2006). c-SRC is a transmembraneous regulator, which plays a role in focal adhesion complexes and cytoskeletal dynamics and mediates proliferatition via the platelet-derived growth factor receptor (PDGF-R) (Brown and Cooper, 1996; Yamana et al., 2006; Huveneers and Danen, 2009). Furthermore, pro-oncogenic properties make c-SRC an important target in cancer research (Musumeci et al., 2012; Gargalionis et al., 2014; Varkaris et al., 2014).

The regulation of the downstream signaling of RhoA via either ROCK or mDia1/c-SRC plays an important role in the dynamics and contractility of intracellular stress fibers. While stimulation of the RhoA/mDia1/c-SRC-axis leads to actin polymerization, the stimulation of the RhoA/ROCK-axis leads to actomyosin contractility and inhibits actin depolymerization (Takai et al., 2001; Quack et al., 2009). In migration and contraction of activated HSCs the signaling via RhoA/ROCK is well-investigated. However, little is known about the role of c-SRC and the interplay with RhoA in liver fibrosis in general and in activated HSCs in particular.

In this work, we show for the first time the changes exerted by liver fibrosis in the expression of activating and inactivating phosphorylation sites of c-SRC in both human and rat liver samples. Furthermore, we demonstrate that c-SRC plays a decisive role in RhoA and cytoskeletal protein activity by inhibition of c-SRC in cultivated human hepatic stellate cells.

## Materials and methods

### Animals

All animal testing was carried out using wild type rats. Our studies were approved by the committee responsible for animal studies in North Rhine-Westphalia (LANUV reference number 84-02.04.2014.A137).

### Cholestatic model of fibrosis

Bile duct ligation (BDL) was performed in rats with an initial body weight between 180 and 200 g as previously described (Heller et al., 2003). Sham-operated rats served as controls. Experiments were performed after a 4 week interval to allow development of liver fibrosis.

### Toxic model of fibrosis

Rats with an initial body weight between 80 and 100 g were administered carbon tetrachloride (CCl_4_) via inhalation for 14–16 weeks as described previously (Granzow et al., 2014). Age-matched rats who did not receive CCl_4_ served as controls. Additionally, rats with an initial body weight between 200 and 250 g were orally administered thioacetamide (TAA) weekly for 18 weeks as described previously (Verbeke et al., 2014).

### Tissue collection

After induction of liver fibrosis, the rats were anesthetized and laparotomy was performed for tissue collection. The livers were cut into fragments and stored at −80°C until they were used for qRT-PCR and western blot analysis as described previously (Trebicka et al., 2008, 2010).

### Human liver samples

Human liver samples were taken during liver transplantation from patients with alcohol-induced cirrhosis. Liver samples from non-cirrhotic patients who underwent liver resection served as controls. No patient or donor received catecholamines, angiotensin receptor antagonists or ACE inhibitors prior to transplantation. All samples were snap frozen after excision. The use of human liver samples was approved by the Human Ethics Committee of the University of Bonn (reference number 029/13). All subjects gave written informed consent in accordance with the Declaration of Helsinki.

### Isolation of primary hepatocytes and hepatic stellate cells

Primary rat hepatocytes and hepatic stellate cells were isolated and cultured as described previously (Herman et al., 1988; Wojtalla et al., 2012; Granzow et al., 2014). Viability and purity were routinely more than 95%. For early activation of HSCs, cells were harvested at day 10 and for advanced activation with differentiation to myofibroblast-like phenotype, cells were harvested at third passage.

### Cell culture

Snap-frozen LX2 cells and primary rat hepatic stellate cells were incubated with cell culture medium (DMEM + 20% FBS + Penicillin/Streptomycin) in 250 ml plastic flasks at 37°C. After reaching 80% confluency, cells were passaged with a 1:3 split ratio. Detachment was achieved by incubating the cells with 0.05% Trypsin/EDTA solution (solved in PBS) for 5 min at 37°C. Before transfection, LX2 cells were incubated with transfection media (DMEM with 10% FBS) for 24 h. Plasmids with wildtype RhoA, constitutively active RhoA and dominant negative RhoA were kindly provided by Prof. Dr. Fürst (Institute for Cell Biology, University of Bonn, Germany). Fifteen microliter of the respective plasmid and 37.5 μl of lipofectamine were then incubated for 20 min with 3.6 ml transfection media. Cell media was then aspirated and the cells were incubated with the plasmid/lipofectamine mix, which was carefully added drop-wise. After 4 h, cells were again incubated in transfection media and harvested 3 days later. Efficacy of transfection was tested using qRT-PCR.

### Inhibition of c-SRC

For inhibition of c-SRC 4-Amino-3-(4-chlorophenyl)-1-(t-butyl)-1H-pyrazolo[3,4-d]pyrimidine (PP2; Sigma-Aldrich, Munich, Germany) has been used to blunt c-SRC mediated effects (Hanke et al., 1996; Yoshizumi et al., 2000). Ten micrometer of PP2 were added to the cell culture medium and cells were incubated for 2 days before harvesting.

### Polyacrylamide gels

Either snap-frozen LX2 cells or isolated rat hepatocytes were seeded on fibrinogen-coated polyacrylamide (PAA) gels of variable stiffness. Matrices were prepared on 12 mm cover slips in 6-well cell culture plates as previously described (Olsen et al., 2011). Briefly, gels were cross-linked using a Spectroline Microprocessor controlled UV Cross-linker (Thermo Scientific, Waltham, USA). Prepared gels were then coated with fibronectin (Sigma-Aldrich, Munich, Germany). Healthy parenchymatous conditions were simulated by soft matrices with 1 kPa shear modulus, while matrices used to simulate stiff conditions had a shear modulus of 12 kPa. Cell culture was then performed on these gels as described above.

### qRT-PCR

Liver homogenates from either fibrotic or non-fibrotic rats were prepared using previously described methods (Trebicka et al., 2010). RNA was isolated from samples using the Qiazol reagent as instructed by the manufacturer (Qiagen, Hilden, Germany) (Trebicka et al., 2013; Anadol et al., 2015). The following assays provided by Applied Biosystems (Foster City, USA) were used: *ACTA2* (αSMA, Hs00426835_g1), *COL1A1* (Hs00164004_m1), *Src* (Rn01418228_m1) *PDGFRB* (Hs01019589_m1), *RHOA* (for human; Hs01051295-m1), and *RhoA* (for rat; Rn04219609_m1). *Albumin* (Rn-Alb_1_SG) was provided by Qiagen (Hilden, Germany). Samples were normalized to 18s rRNA.

### Western blotting

Snap-frozen cells and liver samples were processed as previously described using sodium dodecyl sulfate polyacrylamide gel electrophoresis (SDS-PAGE) gels and nitrocellulose membranes (Kwiecinski et al., 2011). Equal protein loading was assured using Ponceau-S staining. GAPDH served as endogenous control of protein expression. Membranes were incubated with rabbit-anti-p-c-SRC (Tyr418) from Invitrogen (Darmstadt, Germany), rabbit-anti-c-SRC, rabbit-anti-p-c-SRC (Tyr530), mouse-anti-RhoA, and rabbit-anti-GAPDH primary antibodies and corresponding peroxidase-coupled secondary antibodies from Santa Cruz Biotechnology (Heidelberg, Germany). Results were analyzed using Chemi-Smart digital detection (PeqLab, Biotechnologies, Erlangen, Germany) after enhanced chemiluminescence (ECL, Amersham, UK).

### Statistical analysis

Group size was at least *n* = 5 for each group. Graphs are presented as means ± standard deviation and *p* < 0.05 were considered statistically significant. Western blots were measured using digital densitometry software (Bio-1D v.15.02, Vilber Lourmat, Marne-la-Vallée, France) and the respective density of each band was calculated. The fibrosis groups were tested for significance to their corresponding controls using Mann-Whitney *U* test. In qPCR experiments, 2^−ddCT^ was calculated and normalized to the respective control group. Plotting of diagrams and statistic analysis were performed using GraphPad Prism version 4.00 for Windows (GraphPad Software, La Jolla, California, USA).

## Results

### RhoA and c-SRC crosstalk in hepatocytes

**Figure 1:**
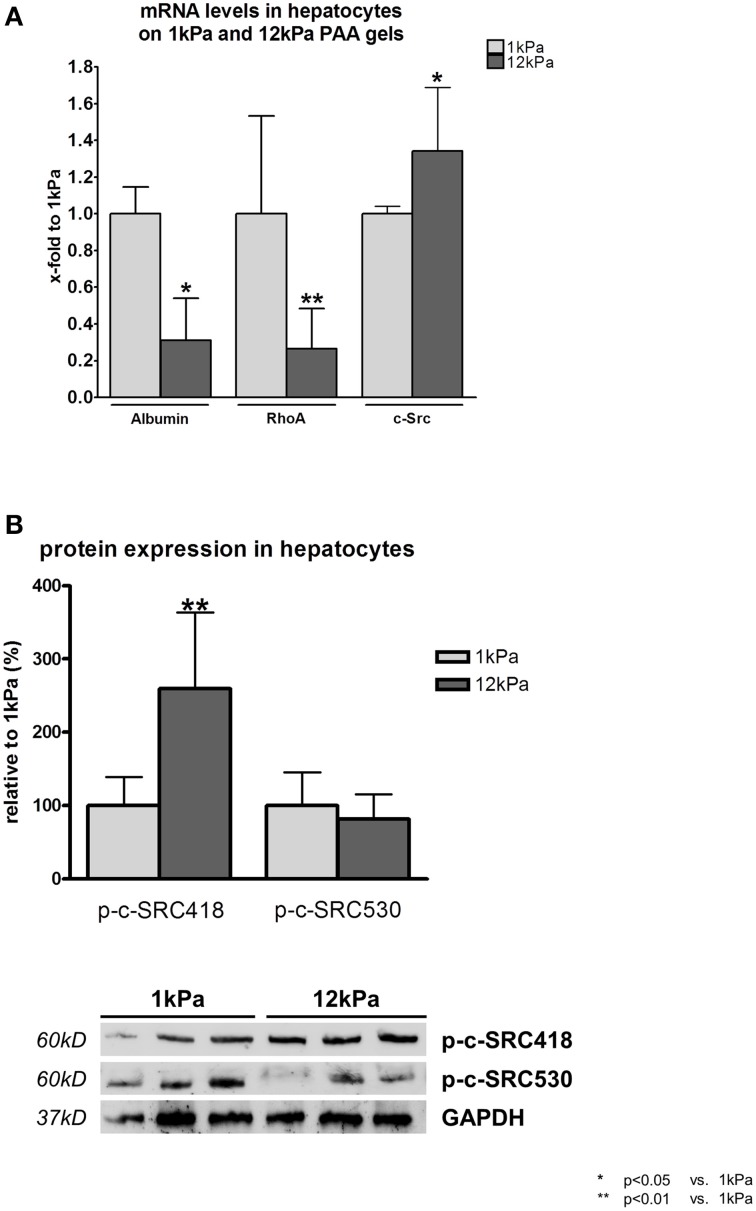
**RhoA and c-SRC crosstalk in hepatocytes**. **(A)** Hepatocytes were incubated on gels with a shear modulus of 1 kPa, simulating healthy liver tissue, and with a shear modulus of 12 kPa, simulating fibrotic tissue. Hepatocyte function was reduced under stiff conditions as shown by *albumin* transcription levels. Transcription of *RhoA* was decreased, while *c-Src* mRNA was increased under stiff conditions. **(B)** Activating phosphorylation (p-c-SRC418) of c-SRC was increased, while inactivating (p-c-SRC530) was decreased under stiff conditions in hepatocytes.

Rat hepatocytes, which were cultivated on PAA gels with a shear modulus of 12 kPa, simulating stiff liver tissue, showed reduced function marked by a significant decrease in transcription levels of albumin compared to hepatocytes cultivated on 1 kPa gels (Figure 1A). Reduced hepatocyte function further led to a significant downregulation of *RhoA* transcription under stiff conditions, while mRNA levels of *c-Src* were increased in these cells (Figure 1A).

Besides transcription of c-Src, also the activation of the c-SRC protein was altered in hepatocytes cultivated on PAA gels with 12 kPa. Phosphorylation at tyrosine 488 (p-c-Src418), the c-SRC activating phosphorylation site, was significantly increased and phosphorylation at tyrosine 530, the c-SRC inactivating phosphorylation site, showed a trend to be decreased under stiff conditions (Figure 1B).

### RhoA and c-SRC crosstalk in human derived HSC cell line LX2 under stiff conditions

**Figure 2:**
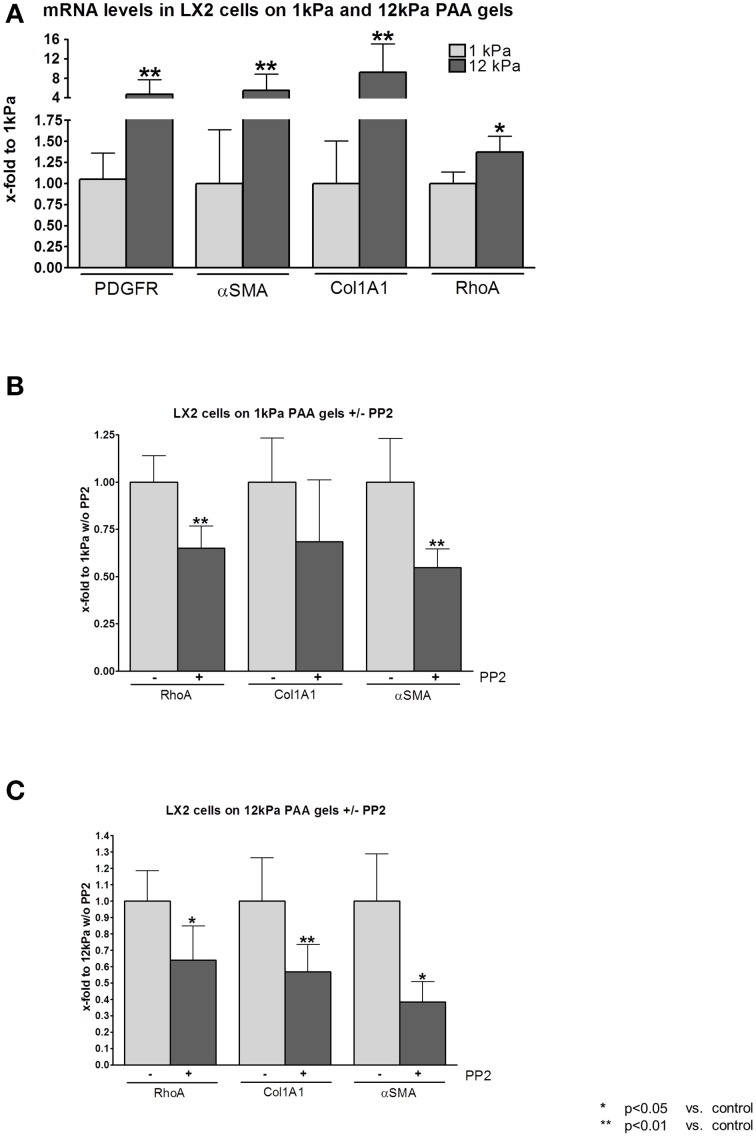
**RhoA and c-SRC crosstalk in human derived HSC cell line LX2 under fibrotic conditions**. **(A)** Human derived hepatic stellate cell line (HSC) LX2 were incubated on gels with shear modulus of 1 kPa, simulating healthy liver tissue, and with shear modulus of 12 kPa, simulating fibrotic tissue. Transcription of proliferative (PDGF-R) and activation (αSMA) markers was increased under stiff conditions. Stiff conditions stimulated LX2 cells to increase collagen production. RhoA was slightly increased by mRNA levels under stiff conditions. **(B)** PP2 administration reduced RhoA, Collagen 1 and αSMA mRNA levels under soft conditions. **(C)** Similarly, in LX2 cell incubated on 12 kPa gels, PP2 administration led to decreased transcription of RhoA, Collagen 1 and αSMA. However, the effect of PP2 in LX2 cells on 12 kPa gels was more pronounced than in LX2 cells on 1 kPa.

In contrast to hepatocytes, cultivation on PAA gels with shear modulus of 12 kPa led to stimulation and activation of hepatic stellate cells (HSC). Both, proliferation as shown by transcriptional levels of *Pdgf-r*, as well as activation and collagen secretion as shown by the surrogate marker α*Sma* and *collagen 1* mRNA levels, were increased when cells were cultivated on PAA gels with stiffness of 12 kPa compared to cells cultivated on PAA gels with stiffness of 1 kPa (Figure 2A). Furthermore, transcription of *RhoA* was increased significantly, but less pronounced, in LX2 cells under stiff conditions (Figure 2A).

Incubation with PP2, a selective inhibitor of the c-SRC tyrosine kinases, decreased transcriptional levels of *RhoA* and of the HSC activation markers *collagen 1* and α*Sma* even under soft conditions when LX2 cells were cultivated on PAA gels with shear modulus of 1 kPa (Figure 2B). On PAA gels with 12 kPa stiffness incubation with PP2 revealed similar results compared to the experiments performed on 1 kPa gels. However, the effect of c-SRC inhibition by PP2 on reduction of *RhoA, collagen 1*, and α*Sma* mRNA levels was stronger in LX2 cells cultivated on 12 kPa PAA gels (Figure 2C).

### RhoA and c-SRC crosstalk in hepatic stellate cells under extremely stiff conditions

**Figure 3:**
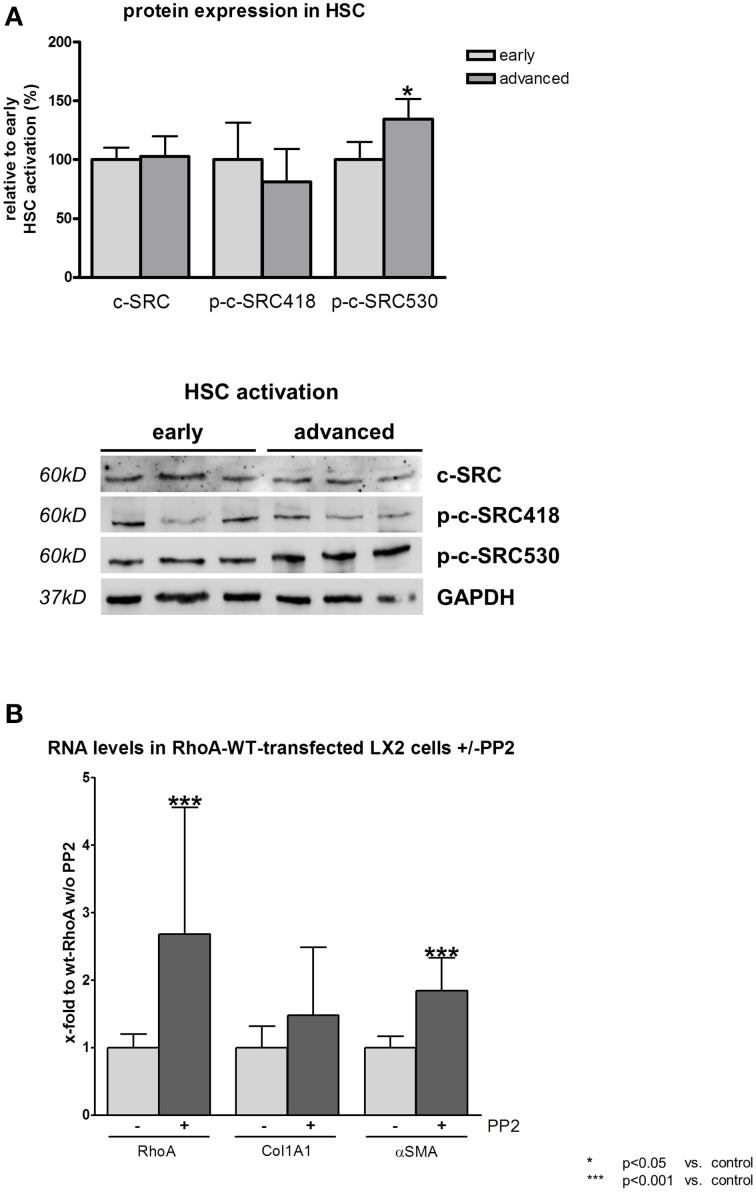
**RhoA and c-SRC crosstalk in hepatic stellate cells under cirrhotic conditions. (A)** Primary rat hepatic stellate cells (HSC) were cultivated on plastic, simulating extremely stiff conditions. For early activation of HSCs, cells were harvested at day 10 and for advanced activation to myofibroblast-like phenotype, cells were harvested at third passage. Inactivating phosphorylation at tyrosine 530 of c-SRC was increased in activated myofibroblast-like HSCs, while activating phosphorylation at tyrosine 418 of c-SRC showed a trend toward a decrease. Overall c-SRC protein did not change upon HSC activation progress. **(B)** Inhibition of c-SRC by PP2 led to increased mRNA levels of RhoA, Collagen 1 and αSMA in activated myofibroblast-like HSCs.

The high matrix stiffness prevailing in plastic cell culture flasks simulates the extremely stiff matrix conditions in liver cirrhosis. Under these conditions the c-SRC activation in primary rat HSCs drops with progressive HSC activation as shown by a significant increase in p-c-SRC530 and a decrease in p-c-SRC418 (Figure 3A). Total protein levels of c-SRC remained unchanged upon progressive HSC activation under extremely stiff conditions (Figure 3A).

Inhibition of c-SRC by PP2 under extremely stiff conditions led to a significant upregulation of *RhoA* mRNA (Figure 3B). Furthermore, PP2 significantly increased HSC activation as demonstrated by α*Sma* mRNA levels. Additionally, also collagen production showed a trend toward an increase in transcriptional level (Figure 3B).

### RhoA and c-SRC crosstalk in experimental and human liver cirrhosis

**Figure 4:**
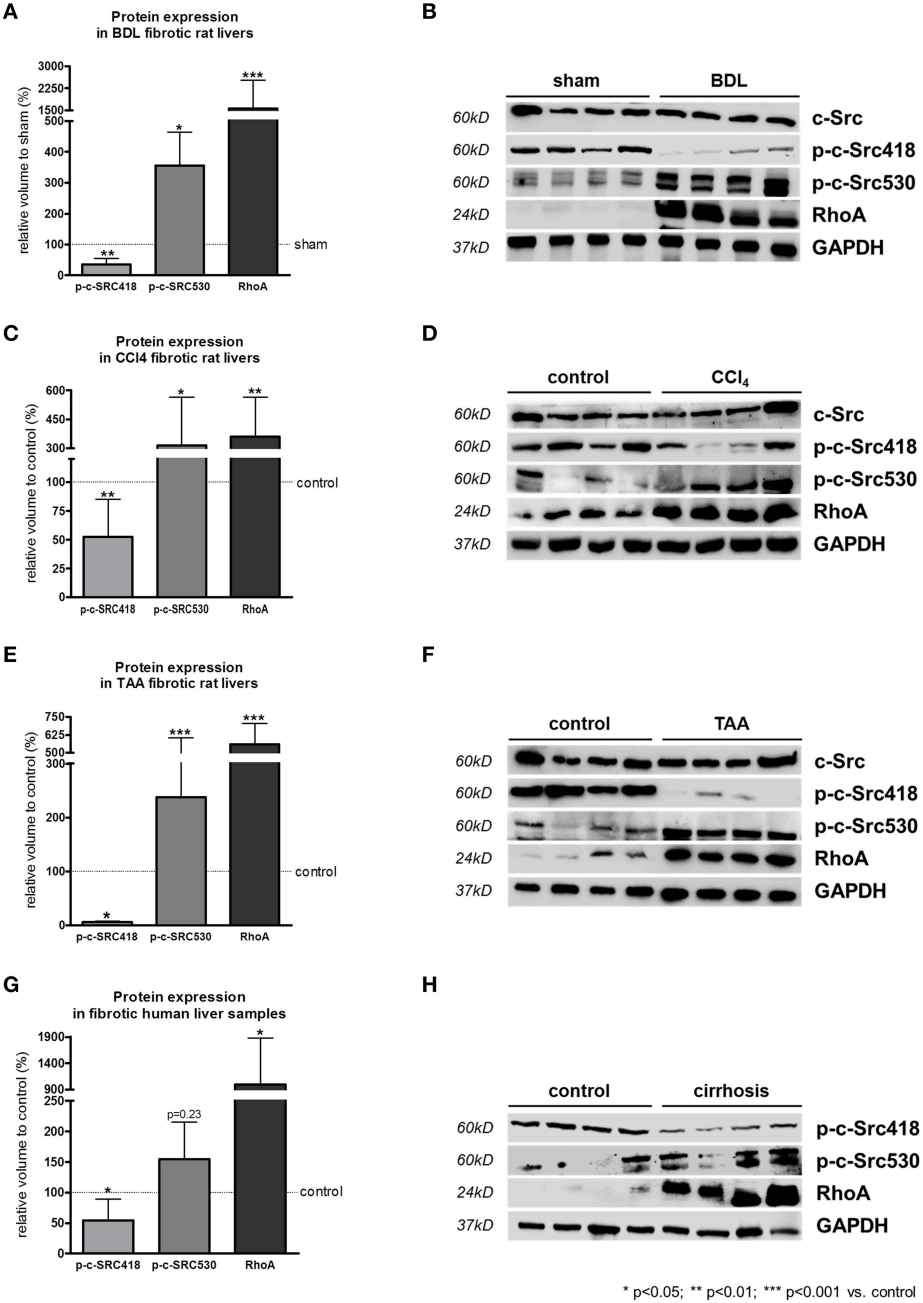
**RhoA and c-SRC crosstalk in experimental and human liver cirrhosis**. Protein expression of phosphorylated c-SRC and RhoA in cholestatic BDL **(A,B)**, toxic CCl_4_ **(C,D)**, or TAA **(E,F)** intoxication liver cirrhosis models, as well as in samples of human liver tissue **(G,H)** showed increased inactivation of c-SRC (p-c-SRC530) in liver cirrhosis. In contrast, activating phosphorylation (p-c-SRC418) was decreased. RhoA was highly upregulated in experimental and human liver cirrhosis. Controls were sham operated rats for the BDL model, untreated rats for CCl_4_ and TAA model an liver biopsies from non-cirrhotic patients.

In bile duct ligated (BDL) rats, a model for cholestatic liver cirrhosis, the inactivating phosphorylation at tyrosine 530 of c-SRC was significantly increased compared to sham operated rats (Figures 4A,B). As a consequence, phosphorylation at tyrosine 418 of c-SRC was significantly decreased in these animals, while total protein levels of c-SRC remained unchanged upon BDL (Figures 4A,B). In addition, RhoA was highly upregulated in BDL rats (Figures 4A,B).

Carbon tetrachloride (CCl_4_) and thioacetomide (TAA) intoxication are both models for toxic liver cirrhosis. Parallel to the results observed in BDL rats, phosphorylation at tyrosine 530 of c-SRC was significantly increased, while phosphorylation at tyrosine 418 of c-SRC was significantly decreased in CCl_4_ (Figures 4C,D) and TAA (Figures 4E,F) intoxicated rats compared to untreated control rats. Also in experimental models of toxic liver cirrhosis levels of total c-SRC remained unchanged, while RhoA was significantly increased (Figures 4C–F).

Changes in c-SRC activation in models of experimental liver damage mirrored the situation in human liver cirrhosis. In liver samples of cirrhotic patients c-SRC activating phosphorylation at tyrosine 418 was significantly downregulated compared to non-cirrhotic control liver samples (Figures 4G,H). As a consequence c-SRC inactivating phosphorylation at tyrosine 530 was increased in liver samples of cirrhotic patients. As shown for experimental liver cirrhosis, also in human liver cirrhosis RhoA expression is highly upregulated compared to the non-cirrhotic control liver samples (Figures 4G,H).

**Figure 5:**
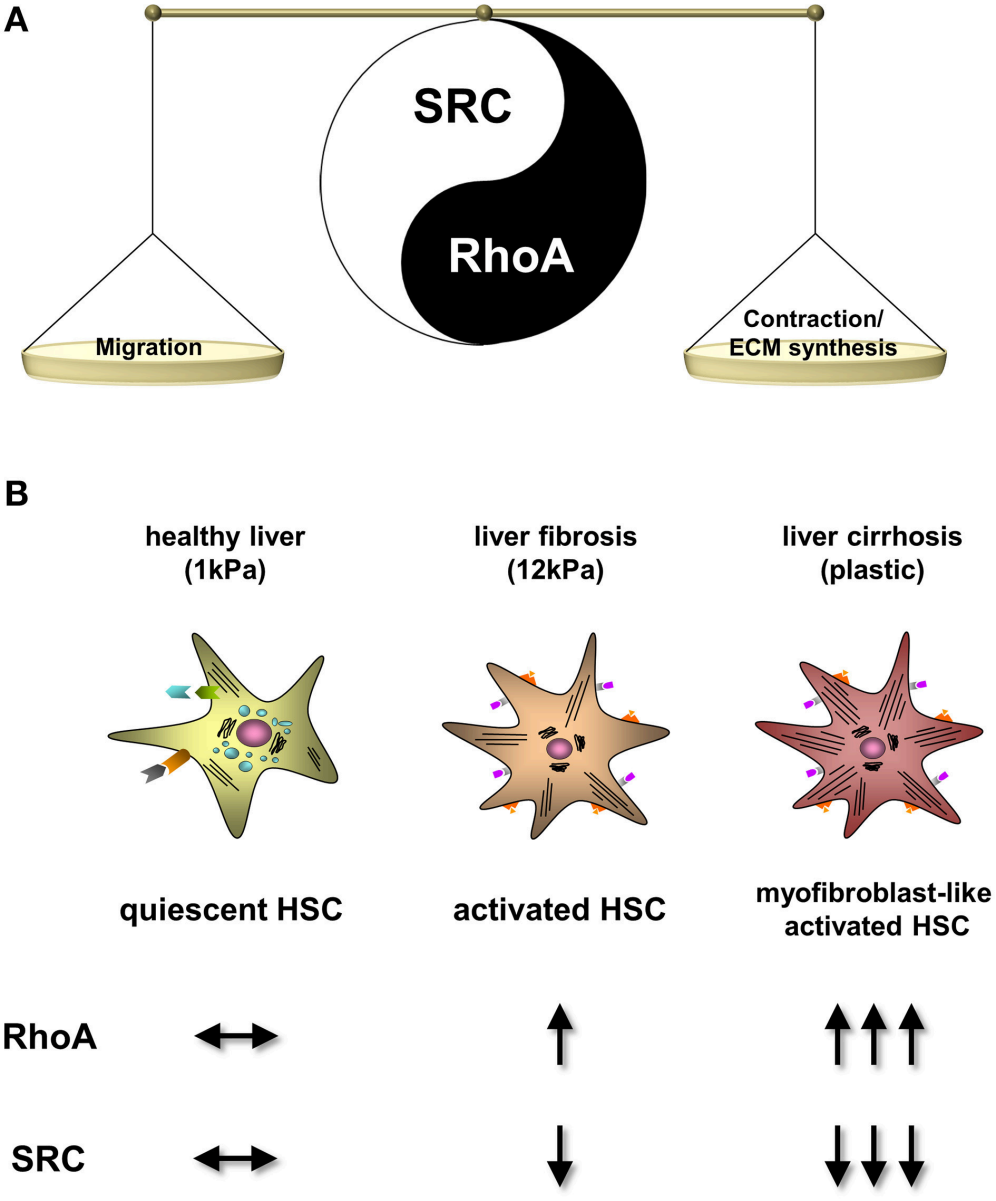
**Regulation of RhoA and c-SRC activity upon progression of liver fibrosis. (A)** Scheme of the RhoA and c-SRC crosstalk in hepatic stellate cells (HSC). c-SRC activation primary stimulates migration of HSCs and activation of RhoA leads to contraction and collagen production. Therefore, c-SRC seems to play a important role in early stages of fibrosis, when HSCs are proliferating and migrating. In contrast, RhoA plays a pivotal role in progressed fibrosis, when HSC phenotype changes into a myofibroblast-like phenotype. **(B)** Progression of HSC activation into myofibroblast-like cells and the corresponding cell culture conditions mimicking fibrosis progression. Upon HSC activation RhoA becomes highly upregulated, while c-SRC activation is suppressed.

These data suggest a marked counterplay of RhoA and c-SRC expression in human and experimental liver cirrhosis (Figure 5A).

## Discussion

In the present study, we demonstrate for the first time that the activity of c-SRC decreases with progressive liver fibrogenesis and hepatic stellate cell (HSC) activation (Figure 5B). We could show that this effect on c-SRC is regulated by a counterplay with RhoA, which, in contrast, is upregulated with progressing HSC activation (Figure 5A). Thereby, c-SRC inactivation was reached by increased phosphorylation at tyrosine 530 and decreased phosphorylation at tyrosine 418, while total protein levels of SRC in HSCs were unchanged upon progressing liver damage. Since this crosstalk of RhoA and c-SRC could be observed in cholestatic and toxic models of liver cirrhosis, as well as in alcohol-induced human liver cirrhosis, our data suggest that the mechanisms are independent of the etiology.

SRC consists of four SRC homology (SH) domains and phosphorylation at tyrosine 530 on SH2 leads to inactivation of SRC. Dephosphorylation at tyrosine 530 by different kinases, such as SH2-containing phosphatases, allows autophosphorylation at tyrosine 418/419 on SH1, which includes the kinase domain, and leads to activation of SRC (reviewed in Yeatman, 2004). Members of the SRC family kinases have been broadly investigated in cancer due to their pro-oncogenic characteristics (Musumeci et al., 2012; Gargalionis et al., 2014; Varkaris et al., 2014). While first results for c-SRC targeting have been reported in the treatment of idiopathic pulmonary fibrosis, systemic sclerosis and glioblastoma (Beyer and Distler, 2013; Ceccherini et al., 2015), its role in liver fibrosis progression is not yet understood.

To simulate either healthy or fibrotic environments, we chose to incubate hepatocytes and hepatic stellate cells on polyacrylamide matrices of a defined shear modulus, since it has been shown that a stiff environment is required for differentiation of HSCs to a myofibroblastic phenotype (Olsen et al., 2011). Plastic cell culture flasks are extremely stiff and out of the physiologic range, but provide a useful model system to mimic cells receiving maximal stimulation from a stiff matrix. Hepatic stellate cells that grow in these containers can often only be observed in a highly activated and fully transdifferentiated myofibroblastic state. Results obtained from cells incubated on plastic are therefore restricted to describe only end-stage liver disease. Polyacrylamide matrices allow a better analysis of cells, since different stages of fibrosis progression can be simulated under more physiologically relevant conditions (Olsen et al., 2011).

Inhibition of c-SRC by PP2 as a therapeutic approach may be promising only in early fibrotic stages, when HSCs are migrating. In this stage c-SRC is still active and drives cytoskeletal dynamics and cell motility by stimulation of actin polymerization (Takai et al., 2001; Quack et al., 2009). Furthermore, c-SRC is increased in monocytes and macrophages and mediates secretion of pro-inflammatory cytokines (Sarang et al., 2011; Yokoi et al., 2011), which may contribute to HSC activation upon liver damage.

In contrast, in progressive liver disease activated HSCs develop a myofibroblast-like phenotype. Activated myofibroblast-like HSCs are contractile and drive hepatic fibrogenesis (Friedman, 2003; Mederacke et al., 2013). These effects are mediated by upregulated RhoA/Rho-kinase signaling upon liver damage (Trebicka et al., 2007, 2010). Our group has demonstrated several times that the signaling cascade via JAK2, RhoA, and Rho-kinase signaling is upregulated in liver fibrosis and located mainly in myofibroblast-like activated HSC (Zhou et al., 2006; Granzow et al., 2014; Klein et al., 2015). With the current study we show that increased RhoA activity leads to decreased c-SRC activity with progressive HSC activation. At this stage, inhibition of c-SRC by PP2 failed to improve transcription of fibrogenic markers, probably since c-SRC activity is already low under these conditions, due to the increased phosphorylation at tyrosine 530. This data suggests that the phenotype change of HSC is mediated by a crosstalk of RhoA and c-SRC.

ECM components are responsible for increased matrix stiffness in liver fibrosis and may influence expression of c-SRC via growth factor or hyaluronan receptors (e.g., CD44) or focal adhesion complexes (Nikitovic et al., 2013). Furthermore, ECM components may have direct paracrine and endocrine effects on HSC function and intracellular signaling in liver fibrosis (reviewed in Wells, 2013; Karsdal et al., 2015). Besides increased matrix stiffness, which is only one factor of liver fibrosis, the crosstalk of RhoA, and c-SRC may be triggered by other factors such as proinflammatory or profibrotic cytokines upon liver disease progression. Also regulatory miRNAs in response to inflammatory stimuli could play a role. However, the molecular mechanisms which regulate the crosstalk of RhoA and c-SRC remain unclear and should be investigated in future studies.

Besides inhibition of c-SRC, PP2 has been described to have a weak affinity to inhibit other kinases like JAK2 (Hanke et al., 1996), which is an upstream regulator of RhoA activity (Granzow et al., 2014; Klein et al., 2015). However, the doses of PP2 used in our *in vitro* experiments was very low and much higher doses would be of need to inhibit the JAK2/RhoA axis (Hanke et al., 1996).

In conclusion, this study provides insight into the role of matrix stiffness on c-SRC activity and the crosstalk of RhoA and c-SRC upon progressing liver damage. Furthermore, the usage of PAA gels of different elasticities, simulating different stages of fibrosis progression, proves to be useful for *in vitro* experiments to investigate molecular and pathomechanistic changes triggered by liver fibrosis progression.

## Author contributions

JG, RS designed the original study, wrote the first draft of the article and acquired, analyzed, and interpreted the data. JB, SK, FU acquired, analyzed, and interpreted the data. PV, DF, WL, JP provided substantial material and methods and interpreted data. CS provided administrative support and interpreted data. JT designed the original study, interpreted the data wrote the first draft of the article, provided administrative support and supervised the study. All authors commented on the drafts of the article and approved the final article.

## Funding

The study was supported by grants from Bonner Forum for Biomedicine (to JT), the Deutsche Forschungsgemeinschaft (SFB TRR57), as well as from grants of H. J. and W. Hector Stiftung (to JT).

### Conflict of interest statement

The authors declare that the research was conducted in the absence of any commercial or financial relationships that could be construed as a potential conflict of interest.
